# Genomic insights into monensin resistance development in *Eimeria tenella*

**DOI:** 10.3389/fvets.2025.1459791

**Published:** 2025-04-01

**Authors:** Xiaolong Gu, Sufang Fang, Hongbin Liu, Yubo Shi, Yuanyuan Zhang, Peng Wang, Ping Cui, Xinming Tang

**Affiliations:** ^1^College of Animal Science and Technology, Hebei North University, Zhangjiakou, China; ^2^College of Veterinary Medicine, China Agricultural University, Beijing, China; ^3^School of Pharmacy, Hebei North University, Zhangjiakou, China; ^4^Key Laboratory of Animal Biosafety Risk Prevention and Control (North) and Key Laboratory of Veterinary Biological Products and Chemical Drugs of MARA, Institute of Animal Sciences, Chinese Academy of Agricultural Sciences, Beijing, China; ^5^College of Biological Sciences, China Agricultural University, Beijing, China

**Keywords:** *Eimeria tenella*, monensin, resistance pressure, experimental evolution, genome resequencing

## Abstract

**Introduction:**

Monensin resistance in *Eimeria tenella* poses a significant challenge in poultry farming, compromising the effectiveness of this widely used anticoccidial drug. The present study aimed to identify candidate mutated genes in *Eimeria tenella* associated with monensin resistance through experimental evolution and pooled genome sequencing.

**Methods:**

The monensin-resistant (MR) strains were rapidly generated by 6 generations of serial passage under gradient monensin treatments using Houghton strain as the parental strain. Genomic sequencing was applied to uncover genetic changes during passages under drug selective pressure. Comparative analysis between resistant and control populations was performed by using the ΔSNP-index and F_ST_ values to identify loci with significant selective sweeps. Stringent thresholds were applied to pinpoint candidate genes, followed by annotation and analysis of their potential functions.

**Results and discussion:**

The genetic diversity of MR parasites remained stable across generations, despite varying drug concentrations. Seven candidate genes with 11 missense mutations were identified in MR strains. Key genes include ETH2_0729200 (dynein motor protein), ETH2_0729400 (esterase/lipase), and ETH2_0730000 (pyridine nucleotide-disulfide oxidoreductase) annotated in both the selective sweeps by using ΔSNP-index and F_ST_ methods. Further experimental validation of these candidate genes is essential to elucidate their roles in monensin resistance. This research contributes valuable insights into the molecular basis of resistance pressure in *Eimeria* parasites, potentially informing future strategies for the control of coccidiosis.

## Introduction

Chicken coccidiosis is a disease caused by one or various species of *Eimeria* that severely harms the growth and development of poultry, resulting in significant economic losses to the poultry industry (1). The disease not only leads to widespread illness and death among chickens but also hinders their growth and development, reducing feed efficiency (2, 3). The widespread prevalence of coccidiosis is primarily due to the direct developmental cycle of *Eimeria*, with chickens being their only host (4). The oocysts of *Eimeria* have an extremely strong resistance, and conventional sanitation and epidemic prevention measures cannot prevent the spread of *Eimeria* oocysts. Moreover, intensive farming practices create favorable conditions for coccidiosis outbreaks (4). With the long-term use of anticoccidial drugs, the issue of resistance pressure in chicken *Eimeria* has become increasingly common (5). Once *Eimeria* develops resistance to a particular drug, it can tolerate concentrations four to eight times higher, or even more, than the continuous use concentration. Additionally, it exhibits cross-resistance and multi-drug resistance, which complicates the prevention and control of coccidiosis in chickens (6). Elucidating the genetic loci and mechanisms underlying resistance pressure in *Eimeria* parasites is of significant importance for the development of new anticoccidial drugs and the formulation of effective coccidiosis control strategies (7).

Directed evolution in conjunction with whole-genome sequencing has proven to be a powerful approach for identifying resistance targets and elucidating the pathways of numerous antiparasitic agents, including artemisinin, quinoline, and halofuginone (8, 9). Notably, the prolyl-tRNA synthetase (PRS) enzyme has been confirmed as the molecular target for halofuginone in both *Plasmodium falciparum* and *E. tenella*, as evidenced by studies (10). Monensin, a widely utilized anticoccidial, has been the focus of resistance research. Through experimental evolution and linkage group selection in *Eimeria* species, Zhang et al. identified a total of 16 nonsynonymous mutations in protein-coding genes within monensin-resistant strains (11). However, pinpointing the key proteins that confer resistance to monensin remains a challenge.

In this study, we set out to delineate the loci associated with monensin resistance in *Eimeria* by leveraging experimental evolution and whole-genome sequencing. We developed eight distinct *E. tenella* lines with incremental resistance to monensin through continuous drug exposure and subsequently analyzed selected genomic regions. Additionally, a selective sweep analysis was conducted to pinpoint the genomic regions that are critical for monensin resistance, leading to the identification of seven candidate genes. Our findings contribute foundational knowledge to the understanding of the molecular mechanisms underlying resistance pressure in coccidian parasites.

## Materials and methods

### Animals and parasites

All animal procedures in this study were conducted in strict compliance with the guidelines established by the Institutional Animal Care and Use Committee (IACUC) and were approved by the Administration Committee of Laboratory Animals at Hebei North University.

For the purposes of proliferation and selection of resistance pressure lines, we utilized two-to-four-week-old Hubbard broilers sourced from Yizhou Animal Husbandry Co., LTD in Zhangjiakou, China. The broilers were provided with a coccidia-free diet and had unrestricted access to water. The experimental protocols involving the chickens were conducted in full accordance with the Hebei North University’s Institutional Animal Welfare and Animal Experimental Ethical Inspection standards.

The wild-type *Eimeria tenella* Houghton strain (EtH strain), known for its sensitivity to the anticoccidial drug monensin, was generously provided by Prof. Xun Suo’s laboratory, China Agricultural University. The methodologies for oocyst collection, sporulation, and purification were executed following the established procedures detailed in our previous publication. The monensin-resistant (MR) strains were generated by 6 generations of serial passage under gradient monensin treatments (from 100 to 1,000 ppm [mg/kg]) using EtH as the parental strain (P0).

Additionally, five monensin-sensitive *E. tenella* stains were isolated using single oocyst isolation method from 38 fecal samples collected from 12 distinct farms in four different provinces within China. Two isolates were obtained from two separate farms in Shijiazhuang city, designated as Sjz-1 and Sjz-2. An additional isolate was sourced from Nanjing city, labeled as NJEt. One isolate was obtained from a farm in Xi’an city, denoted as XAEt, and another from a location in Sichuan province, denoted as SCEt.

### Development of resistance pressure in *Eimeria tenella*

In the first trial stage, a total of 400 Hubbard broilers, aged 14 days, were randomly allocated into two experimental groups: the oocyst propagation group (Group 1) and the treatment group (Group 2). On the initial day of the study (Day 0), 10 broilers in Group 1 were orally inoculated with 5,000 viable EtH sporulated oocysts. Concurrently, the broilers in Group 2 were received a diet supplemented with 100 mg/kg monensin throughout the experimental period. From Day 8, feces containing shed oocysts were collected from Group 1 and introduced into the litter of Group 2 until the detection of oocysts in Group 2. Daily measurements of oocyst output per gram of litter were conducted for both groups. On Day 33, oocysts were observed in Group 2’s litter, 10 chickens were systematically removed for oocyst harvesting every 2 days. The oocysts harvested from Group 2 was designated as 1 × P1, which is resistant to 100 mg/kg monensin of passage 1.

In the second trial stage, sporulated oocysts (1 × P1) were subsequently used to infect ten 14-day-old broilers, with a stepwise increase in monensin concentration in their feed: 133 mg/kg for the first 11 days post-inoculation (dpi), followed by 200 mg/kg from 12 to 16 dpi, culminating in 400 mg/kg on Day 17. Thereafter, the progeny parasites from the second trial were propagated in chickens fed with the diet containing 400 mg/kg monensin, and this propagation process was iteratively continued for the subsequent five generations. Following the fifth generation, the propagation was conducted with a monensin concentration of 1,000 mg/kg.

### Oocyst collection and DNA extraction

Fecal oocysts were systematically collected from each bird between 6 and 8 days post-infection during the two experimental trials. The genomic DNA of the oocysts was extracted following the protocol outlined in a previous study (12), and the DNA samples were sequenced using Illumina NovaSeq 6000 platform.

### Genome mapping, variant calling and annotation

Firstly, the Burrows-Wheeler Aligner (BWA) was used to align the clean sequencing read against the reference genome, *Eimeria tenella* Houghton 2021 (GCA_905310635.1), downloaded from Toxodb (https://www.toxodb.org/toxo/app/record/dataset/TMPTX_etenHoughton2021). The Genome Analysis Toolkit (GATK) (13) version 4.0 was then used to call the variants, followed by filtering out the unmapped and non-unique reads. The single nucleotide polymorphisms (SNPs) were further submitted to VCFtools (14) for quality control, high-quality SNPs with less than 10% missing data, a quality score greater than 20, mean depth values between 10 and 1,000, and genotype quality above 20 were kept for subsequent analyses. The protein-coding genes within interested regions or loci were annotated using the BEDTools (15) and Ensembl Variant Effect Predictor (16).

### Data statistics and analysis

To pinpoint genomic regions associated with monensin resistance, we employed the population differentiation index (F_ST_) (17) and the ΔSNP-index to detect signatures of drug selection. The SNP index for each strain were calculated to compare the allele frequency spectrum among the monensin-resistant (MR) samples and monensin-sensitive (MS) samples, primarily to illustrate the changes in mutate allele frequencies across the drug selection generations.

The F_ST_ between MR and MS groups were calculated using Poolseq. The ΔSNP-index between the two groups were calculated as the average SNP index in MR minus the average SNP index in MS. The MR group include six strains, including 10 × P2, 10 × P3-1, 10 × P3-2, 4 × P4, 4 × P5-1, and 4 × P5-2. The MS group include P0 (EtH) and five wild-type isolates from different domestic farms. To prevent the estimates of single locus influenced by sequencing or spurious errors, we smoothed the estimates in sliding 10 kb windows with a step size of 5 kb. The top 1% outliers of windows were regarded as the putative genomic regions under selection.

## Results

### Induction and phenotyping of MR strains

We implemented an experimental evolution strategy aimed at developing MR strains from the EtH. Induction and Phenotyping of MR Strain were written in detail in our research (18).

In the initial experiment, Oocysts were harvested from days 33 to 39 post-inoculation, leading to the isolation of the first monensin-resistant strain, designated as 1 × P1. During the second experiment, a total of eight distinct monensin-resistant strains were isolated from individual birds exposed to a gradient of monensin concentrations (133 mg/kg, 200 mg/kg, 400 mg/kg and 1,000 mg/kg). The strains were labeled as follows: 2 × P1-1, 2 × P1-2, 4 × P4, 4 × P5-1, 4 × P5-2, 10 × P2, 10 × P3-1, and 10 × P3-2, with the notation indicating the strain origin and the generation of passage.

### The sequencing data and detected SNPs

In the present study, we sequenced 14 *Eimeria tenella* samples, generating a panel of clean data ranging from 35 to 74 million clean reads, corresponding to genome coverage ranging from 103× to 215×. Except for the 10 × P2 (alignment rate = 92.67%), the alignment rates of the other samples against the reference *E. tenella* genome were almost greater than 97% (Supplementary Table S1).

**Figure 1:**
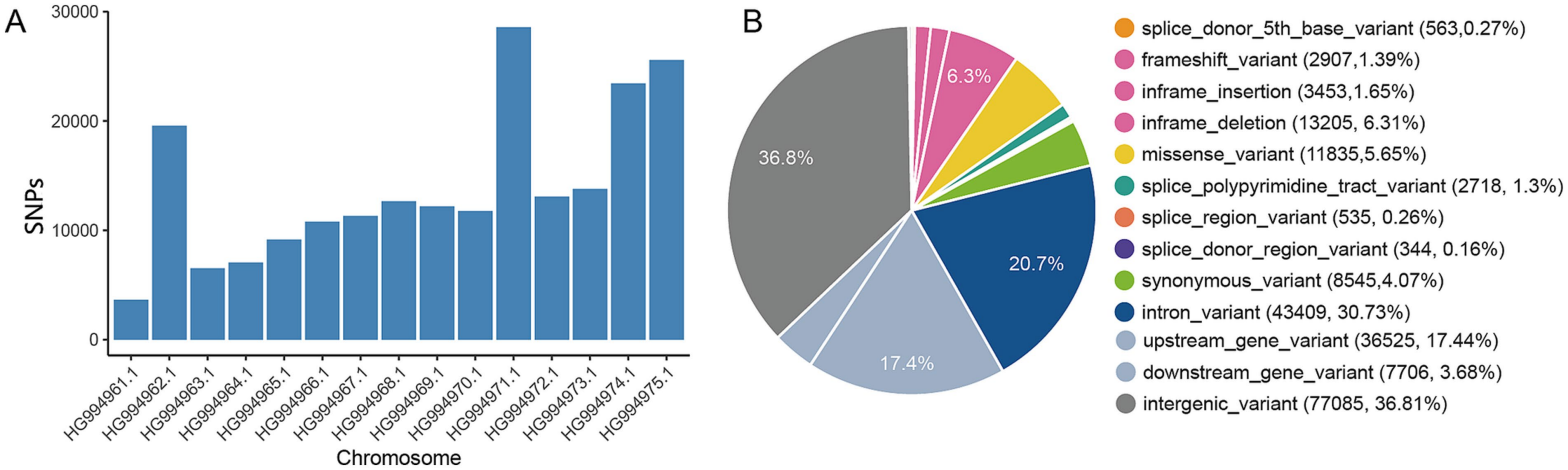
Detection of single nucleotide polymorphisms (SNPs) in *E. tenella* genomes. **(A)** Distribution of SNPs across the chromosomes of *E. tenella*. **(B)** Overview of SNPs annotated using Ensembl Variant Effect Predictor (VEP) version 105.

In total, 209,422 high-quality single nucleotide variants (SNPs) were identified, comprising 77,085 within intergenic regions and 43,409 within intronic regions (Supplementary Table S2). The distribution of SNPs across all chromosomes is depicted in Figure 1A and Supplementary Table S3, with the average marker density per chromosome varying from 16,990 (on chromosome 2) to 2,938 (on chromosome 10) per 1 megabase (Mb) for autosomes (Supplementary Table S3). Exonic variants constituted only 9.72% of the total mutations, which included 11,835 missense and 8,545 synonymous variants (Figure 1B). The statistical summary of SNPs in samples were shown in Supplementary Tables S1–S5.

### The allelic frequency spectrum in MRs

**Figure 2:**
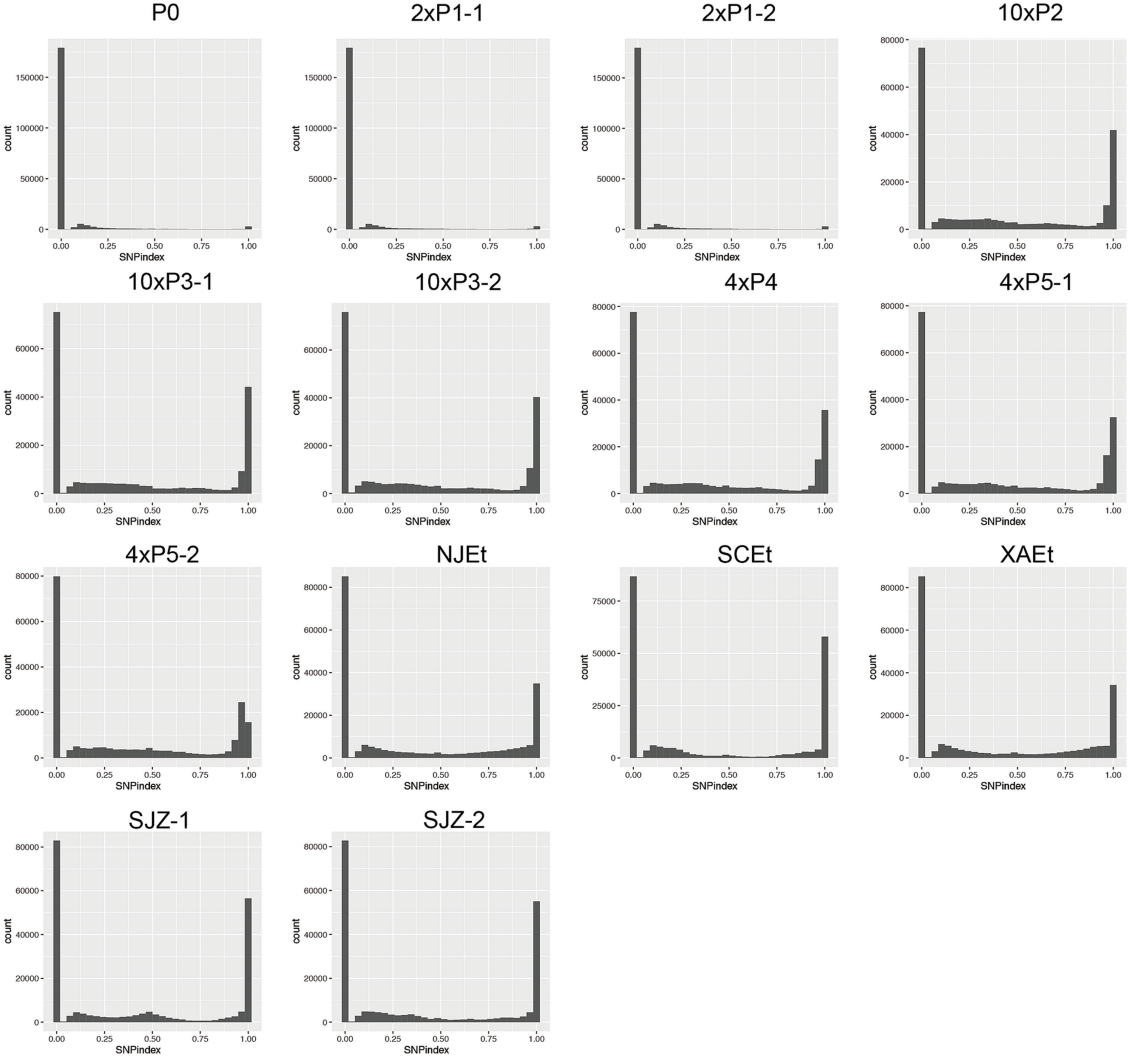
Sliding window plots of delta SNP index across different selected generations of *Eimeria tenella.* The plots display the delta SNP index for various selected generations, calculated using a window size of 10 kb and a sliding size of 5 kb. Each plot shows the widely distributed changes in allele frequency differences between the selected generations (with drug) and the reference population (P0). Progeny oocysts of P0 were designated as P1, Progeny of P1 were designated as P2, etc. P5-1 and P5-2 meant that the oocysts were harvested from different chicken. Resistant oocysts harvested under 100 mg/kg monensin, 200 mg/kg monensin, 400 mg/kg or 1,000 mg/kg in feed were, respectively, designated as 1×, 2×, 4×, or 10×. Five monensin-sensitive *E. tenella* stains were isolated from four provinces within China. Two isolates were obtained from two separate farms in Shijiazhuang city, designated as Sjz-1 and Sjz-2. Three additional isolates were sourced from Nanjing city, Xi’an city and Sichuan province, respectively denoted as NJEt, XAEt and SCEt.

The P0 sample, generated from EtH, is a relatively purified wild-type isolate, exhibited a relatively low number of SNPs compared to the reference genome, over 94% of loci showed the reference alleles in all the parasites within P0 sample, over 4% of loci showed mutated allele in a subset of parasites within P0 sample and the majority of these mutations occurring at a frequency of approximately 0.1, and only 1% of loci showed mutated alleles in all the parasites within P0. The two P1 samples showed similar distribution of SNPs compared to the P0, indicating no significant drug pressure effect on P1. In contrast, the MR samples displayed a significantly higher count of SNPs, with over 50% loci showed mutated alleles in parasites within each sample, respectively (Figure 2). The five wild-type isolates from different domestic farms also exhibited the high abundance of SNPs and allele frequencies.

Nearly half of detected loci acquired mutations under the drug selection from the second generation, the mutated loci for monensin resistance should be present in MRs with a high frequency but absent in P0 and the other sensitive samples. In the P1 samples, the allele frequency of the potential resistance mutation should be very low or undetectable due to the sampling and sequencing errors.

### Genetic diversity of wild-type drug-sensitive *Eimeria tenella* isolates and MRS

**Figure 3:**
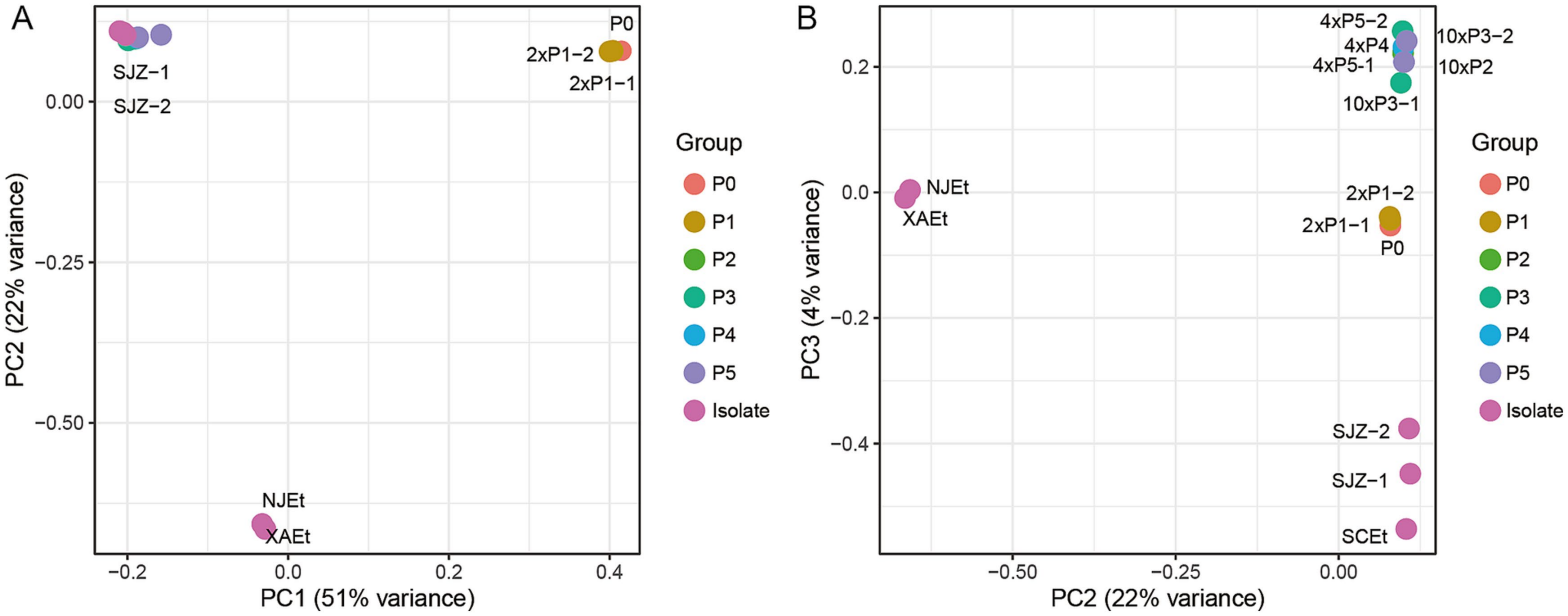
Principal component analysis (PCA) of *E. tenella* based on single nucleotide polymorphisms (SNPs). Principal component analysis (PCA) of all samples in this study. **(A)** PC1, principal components one; **(B)** PC2, principal components two.

The principal component analysis (PCA) was conducted to evaluate the genetic similarities between the samples with varying levels of monensin resistance or sensitivity (Figure 3). The wild-type parent sample P0 and two P1 samples (2 × P1-1, 2 × P1-2) grouped together, which is consistent with the fact the P1 samples generated from P0 and have not yet developed resistance. They are distinct from the MR samples (4 × P4, 4 × P5-1, 4 × P5-2, 10 × P2, 10 × P3-1, 10 × P3-2) and (domestic isolates SJZ-1, SJZ-2, NJEt, XAEt, and SCEt), Based on the PCs, the MR samples cluster together and have been genetic distinguished from the other MS strains, i.e., isolates from different domestic farms and P0 and P1 samples. A high degree of genomic similarity was noted among the MR samples, indicating the MRs under the monensin selection have accumulated similar mutations since the second generation. The allele frequencies changed significantly between the P1 and P2 generations, suggesting the candidate mutation for monensin resistance was initially at a quite low frequency in P1 but rapidly increased during the transition from P1 to P2 due to the drug pressure. The first three principal components explained over 75% of genetic variances among the samples, the MRs generated under 4× and 10× dosages clustered closely, indicating the difference in dosage did not lead to differentiation in their genetic background.

### Identification of candidate loci for monensin resistance

**Figure 4:**
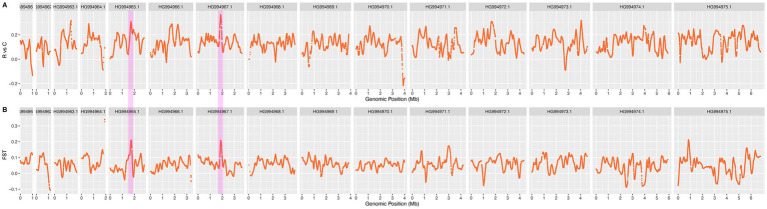
The ΔSNP index and F_ST_ plots for identifying the candidate resistant loci using a sliding window of 10 kb. **(A)** ΔSNP index plot and **(B)** F_ST_ values were generated by comparing all resistant strains with the wild-type strain. Genomic regions associated with resistance were detected using a 10 kb sliding window. The common selected signatures identified by both ΔSNP index and F_ST_ methods on HG994965.1 and HG994967.1 are highlighted with pink bars. In these regions, 11 missense mutations across seven genes were found exclusively in 100% of the resistant strain alleles, while absent in wild-type strains, suggesting their involvement in the resistance phenotype.

The six strains (4 × P4, 4 × P5-1, 4 × P5-2, 10 × P2, 10 × P3-1, 10 × P3-2) of *Eimeria tenella* that demonstrated resistance to a gradient of monensin concentrations (ranging from 100 mg/kg to 1,000 mg/kg) were gathered as a resistance (R) group. To identify the candidate mutation for monensin resistance, the smoothed ΔSNP-index and F_ST_ for every 5 kb window were calculated between MRs and MSs. The P0 and the five wild-type isolates were used as control (C) group. The consistency of the allelic frequency spectrum, releflect by SNP index in MRs (Supplementary Figure S8 in Supplementary material), indicates their genetic similarities, and the genomic diveristy including the resistance loci remained balanced across the generations. Most of the signatures observed in MRs were also present but weakened in ΔSNP-index plot of the R versus C (Figure 4A), these mutations with high frequencies could be random mutations in the initial resistant parasites and heretable to the subsequent generations during the drug selection. The high signatures occurred on varying chromosomes, however, the most highest sites were selected on HG994967.1, which also identified as the candidate region by using F_ST_ (Figure 4B).

### The candidate loci related to the resistance to monensin in *Eimeria tenella*

A comparative analysis was performed between the resistant population and the control population (Figure 4). Four regions on chromosome HG994965.1 and HG994967.1 were identified as candidate regions containing loci with both the ΔSNP-index and F_ST_ values greater than 0.99 quantile thresholds (Figure 4). The shared regions identified in both analyses are hypothesized to be candidate genomic regions for monensin resistance. After excluding synonymous mutation sites, 11 missense mutations within seven genes were found in 100% of the mutated alleles from resistant strains, in contrast to their absence in the wild-type strains (Table 1). ETH2_0729300, ETH2_0729500, and ETH2_0729700 are annotated as conserved hypothetical proteins with no additional functional and structural information available currently. ETH2_0729200 is annotated as a putative gene of dynein motor protein with ATP binding site, ETH2_0729400 is annotated as esterase, and ETH2_0729300 is annotated as pyridine nucleotide-disulfide oxidoreductase. These genes are likely to be functionally important and should be considered in further experimental validation in monensin resistance studies.

**Table 1:** Candidate genes with missense variants for monensin resistance.

DNA	Protein	Gene ID	Product
n.8533G > C	p.Glu2845Gln	ETH2_0729200	GF18580, Dynein heavy chain, N-terminal region 1/Dynein heavy chain, N-terminal region 2, putative, Hydrolytic ATP binding site of dynein motor region D1/AAA domain (dynein-related subfamily), putative
n.562G > A	p.Ala188Thr	ETH2_0729300	Hypothetical protein, conserved
n.1415G > A	p.Gly472Asp	ETH2_0729400	Esterase/lipase domain-containing protein, related
n.239G > T	p.Trp80Leu	ETH2_0729500	Hypothetical protein, conserved
n.1664C > T	p.Ala555Val	ETH2_0729600	Leucine Rich repeat/Leucine Rich Repeat, putative
n.2869A > G	p.Asn957Asp	ETH2_0729600	Leucine Rich repeat/Leucine Rich Repeat, putative
n.5612C > T	p.Ala1871Val	ETH2_0729600	Leucine Rich repeat/Leucine Rich Repeat, putative
n.2561C > T	p.Thr854Ile	ETH2_0729700	Hypothetical protein, conserved
n.6178G > A	p.Ala2060Thr	ETH2_0730000	Pyridine nucleotide-disulfide oxidoreductase/NAD(P)-binding Rossmann-like domain/Flavin containing amine oxidoreductase, putative
n.4747C > A	p.Gln1583Lys	ETH2_0730000	Pyridine nucleotide-disulfide oxidoreductase/NAD(P)-binding Rossmann-like domain/Flavin containing amine oxidoreductase, putative
n.1400 T > C	p.Val467Ala	ETH2_0730000	Pyridine nucleotide-disulfide oxidoreductase/NAD(P)-binding Rossmann-like domain/Flavin containing amine oxidoreductase, putative

## Discussion

In this study, we successfully developed several monensin-resistant strains of *E. tenella* and utilized two forward genetic approaches to map candidate loci associated with monensin resistance and finally identified seven candidate genes within these strains have missense variants in MRs. Our research will contribute to the dissection of the molecular mechanisms of resistance pressure in coccidia.

The generation of resistant strains is essential for understanding the mechanisms underlying resistance pressure in *Eimeria* parasites. Historically, the development of resistant strains of *E. tenella* has been achieved through a dose-escalation strategy that spans numerous generations, such as 20 generations for maduramycin, 18 for diclazuril, and 35 for monensin (19). However, this traditional approach is time-consuming. Sun et al. (20) developed a novel strategy to accelerate the induction of halofuginone-resistant strains of *E. tenella*. And many resistant strains were acquired within just 55 days (20). Sun et al. patented the novel procedure in China. Our study further improved this timeline, obtaining monensin-resistant strains in just 49 days. This novel procedure offers a more efficient means of generating a multitude of resistant strains in a shorter period compared to the previously mentioned methods in Wang et al. (19).

Following gametocyte production by Eimeria parasites, fertilization of macrogametes by microgametes results in the formation of diploid zygotes, which subsequently develop into oocysts. Two types of fertilization occur in this parasite as a result of recombination events between gametocytes of similar genotypes (self-fertilization) or between genetically distinct gametocytes (cross-fertilization). Liu et al. (21) generated two transgenic *Eimeria acervulina* lines to test the rate of cross-fertilization. The frequency of cross-fertilization at an infective dose of 5,000 oocysts is high, around 11% (21). Cross-fertilization generates more opportunities for mutations than self-fertilization. In classical dose-escalation strategy, around 10 birds were used in each generation. And peak oocysts usually were collected. In this study, a population of 200 birds was used to select the resistant oocyst, which generates more opportunities of cross-fertilization between genetically distinct gametocytes to create various mutations. In novel strategy, feces containing shed oocysts were collected from Group 1 from Day 8 to Day 33, then dispersed on the litter of Group 2. During 25 days, at least 3 rounds of life cycle were completed, so feces probably contain the whole oocysts shed in former 2 rounds of life cycle, which introduced more genetic diversity. In classical strategy, the sporulation of the peak oocysts is under the optimum conditions, including temperature, oxygen supply, and moisture. In novel strategy, oocysts in ferment litter will encounter various environment conditions changes, such as temperature, oxygen content, pH and moisture (22). The changes of environment conditions greatly affect the sporulation progress and viability of oocysts, which has influence on oocyst metabolisms, even epigenetics and produce more phenotypic. Above all, novel strategy introduces more phenotypic or genetic diversity, which can generate large numbers of resistant strains in a shorter time.

In Figure 3, we present the results of the Principal Component Analysis (PCA) based on the first three principal components (PCs), which reflect the genetic distances among the samples. In Figure 3A, PC2 (y-axis) does not show significant differences between the groups P0-P5 and the three isolates (SJZ-1, SJZ-2, and SCEt). Additionally, some samples (such as P2 and P4) are not clearly marked in Figure 3A, as they are hidden behind other samples. To better visualize the details, Figure 3B uses PC3 (y-axis) instead, which effectively separates and clusters P0-P5 and the three isolates (SJZ-1, SJZ-2, and SCEt). This adjustment allows each sample to be distinctly represented in the coordinates, providing a clearer view of their relationships.

To uncover the genetic change during the passages under drug selective pressure, the frequency of mutated alleles rapidly increased from 4 × P4 generation, rising from approximately 5% in 2 × P1 to about 56% in 4 × P4 (Supplementary Table S9). Meanwhile, the parasites acquired resistance to monensin. Despite the use of higher drug concentrations across generations, the genetic diversity of parasites from different generations remained stable. Compared with the reference genome, the SNP numbers of all samples range between 74,008 and 81,798 except 2xP1-1 and P0 (Supplementary Table S1).

To elucidate the molecular mechanisms of monensin resistance in coccidia, we employed experimental evolution and pooled genome sequencing strategies to identify candidate genomic regions responsible for this resistance. We have identified seven candidate genes with 11 missense mutations in MRs that may contribute to the monensin resistance (Table 1). Besides four genes (ETH2_0729300, ETH2_0729400, ETH2_0729600, ETH2_0729700) without clear functional annotation, ETH2_0729200 is predicted to encode a dynein motor protein, ETH2_0729400 and ETH2_0930000 are annotated as esterase/lipase and pyridine nucleotide-disulfide oxidoreductase, respectively. ETH2_0729200 is annotated as dynein motor protein, which generally play a critical role in mitosis and cell division. Mutations that affect dynein function could influence cell cycle regulation and growth rates, potentially affecting how cells respond to drug treatments that target rapidly dividing cells. As no known functional annotations are available for the four hypothetical protein-coding genes identified within the candidate loci, we searched for their protein-coding domains using InterPro database. ETH2_0729300 was annotated as a 356-amino acid protein, which may contain an immunoglobulin domain spanning from 111 to 227 amino acids, suggesting a potential role in immune-related functions. ETH2_0729600 was predicted to contain leucine-rich repeat (LRR) domains, which are typically involved in protein–protein interactions, although these domains can serve diverse biological functions. No significant domains were identified in ETH2_0729500 or ETH2_0729700.

Although the mechanisms of resistance to monensin in *Eimeria* are unclear, several potential factors have been proposed. After monensin treatment on *E. tenella* sporozoites, the activity of Na^+^-K^+^ ATPase increases, leading to lactate accumulation and ATP depletion. Monensin acts as a transmembrane sodium carrier, resulting in a net influx of Na^+^, osmotic swelling, and ultimately parasite bursts (23). Augustine et al. (24) demonstrated that resistant sporozoites of *E. tenella* uptake significantly less monensin compared to sensitive sporozoites. Wang et al. (19) found that the membrane fluidity of monensin-resistant *E. tenella* lines was lower than that of sensitive lines, suggesting that changes in membrane composition and fluidity could play a role in resistance. Based on these literatures, monensin resistance in *Eimeria* may involve a combination of reduced drug uptake, alterations in cell membrane properties. Other studies explored the effects of monensin on cell apoptosis. After monensin treatment, the mitogen-activated protein kinase (MEK)-extracellular signal-regulated kinase (ERK) pathway is enhanced to suppress cell proliferation and invasion (25), the levels of CDK6, cyclin D1 and cyclin A decreased, leading to a G(1) and/or a G(2)-M phase arrest and increased cell apoptosis (26), and intracellular oxidative stress is elevated (27). Monensin may have multiple effects on parasites, altering the cell activities and physiology in resistance strains.

One candidate gene identified in the present study, ETH2_0729400, encodes esterase/lipase domain-containing protein. The role of esterase/lipase domain-containing proteins in lipid metabolism is vital as they facilitate the breakdown of complex lipids into simpler molecules, which can be used by the parasite for energy production, membrane synthesis, and signaling pathways (28). A recent study in *Babesia* found an esterase BdFE1, highly conserved among apicomplexan parasites, parasites carrying the L238H mutation in the active site of BdFE1 failed to convert the prodrug to its active moiety and became resistant to the drug (29). Therefore, the ETH2_0729400 may play a critical role in monensin resistance as the potential alterations in lipid metabolic pathways could confer survival advantages to the membrane under drug pressure.

Three missense mutations were found in ETH2_0730000 of MRs, this gene is belonging to the pyridine nucleotide-disulfide oxidoreductases, which is a large and heterogeneous protein family. Pyridine nucleotides, is one of the major redox metabolites, with specific roles in cellular redox homeostasis and the regulation of the cell cycle. Pyridine nucleotide-disulfide oxidoreductase catalyze the pyridine nucleotide-dependent reduction of thiol residues, typically involves the transfer of electrons from NAD(P)H (pyridine nucleotides) to disulfide bonds in substrate proteins. Pyridine Nucleotide-Disulfide Oxidoreductase Domain 2 (PYROXD2) locates to the mitochondrial inner membrane/matrix, knockout of PYROXD2 decreased MMP, intracellular ROS, complex IV activity, cell proliferation, ATP content and mtDNA copy number, but increased mtROS levels and the number of immature mitochondria (30). The mutated ETH2_0730000 could increase the resistance to monensin by alter the electron transmission and redox process in *Eimeria*.

Unfortunately, none of the 16 nonsynonymous mutations reported in the previous study was identified in the present study. In Zhang’s article, *E. tenella* strains resistant to 250 mg/kg of monensin were developed, and experimental evolution coupled with linkage group selection identified 16 nonsynonymous mutations in protein-coding genes within monensin-resistant strains (11). The discrepancy between studies could be due to the use of different resistant strains and the complex mechanism of monensin action, potentially selecting for different resistance loci. Further comparative analysis across multiple studies should be conduct to evaluate the potential candidate genes in different resistant strains. Additional experimental validations of the associated genes are crucial to understanding their roles in resistance pressure in *E. tenella.*

## Data Availability

The sequenced reads are available at the National Genomics Data Center of China (project PRJCA026311), https://ngdc.cncb.ac.cn/search/specific?db=bioproject&q=PRJCA026311.
